# Comparative Efficacy of Transbronchial Needle Aspiration and Cryobiopsies in Thoracic Disorders: A Systematic Review and Meta-Analysis for Optimal Diagnostic Efficacy

**DOI:** 10.3390/life16050768

**Published:** 2026-05-03

**Authors:** Liviu-Ștefan Moacă, Damiana-Maria Vulturar, Daniel-Corneliu Leucuța, Doina Adina Todea, Teodora-Gabriela Alexescu, Maria Adriana Neag, Cezar Aurelian Matau, Anca Dana Buzoianu, Claudia Diana Gherman

**Affiliations:** 1 Department of Surgery-Practical Abilities, “Iuliu Hațieganu” University of Medicine and Pharmacy, Marinescu Street, No. 23, 400337 Cluj-Napoca, Romania; liviu.stef.moaca@elearn.umfcluj.ro (L.-Ș.M.); gherman.claudia@umfcluj.ro (C.D.G.); 2 Interventional Pneumology Unit, Department of Pneumology, University Hospital of Strasbourg, 67091 Strasbourg, France; cezaraurelian.matau@chru-strasbourg.fr; 3 Department of Pneumology, “Iuliu Hațieganu” University of Medicine and Pharmacy, 400332 Cluj-Napoca, Romania; dtodea@umfcluj.ro; 4 Department of Medical Informatics and Biostatistics, “Iuliu Hațieganu” University of Medicine and Pharmacy, 400349 Cluj-Napoca, Romania; 5 4th Department Internal Medicine, “Iuliu Hatieganu” University of Medicine and Pharmacy, 400015 Cluj-Napoca, Romania; teodora.alexescu@umfcluj.ro; 6 Pharmacology, Toxicology and Clinical Pharmacology Department, “Iuliu Hațieganu” University of Medicine and Pharmacy, 400337 Cluj-Napoca, Romania; maria.neag@umfcluj.ro (M.A.N.); abuzoianu@umfcluj.ro (A.D.B.)

**Keywords:** EBUS-TBNA, EBUS-TBMC, diagnostic yield, mediastinal lymphadenopathy, cryobiopsy, meta-analysis, lung cancer

## Abstract

This systematic review and meta-analysis evaluate the comparative diagnostic efficacy and safety of endobronchial ultrasound-guided transbronchial needle aspiration (EBUS-TBNA) and transbronchial mediastinal cryobiopsy (EBUS-TBMC) for sampling mediastinal and hilar lymph nodes. Following the PRISMA 2020 guidelines, 20 studies published between January 2020 and July 2025 were analysed to provide a comprehensive performance overview. The results demonstrate that EBUS-TBMC offers a significantly higher overall diagnostic efficacy compared to EBUS-TBNA, with a pooled risk difference (RD) of 0.30 (95% CI: 0.17–0.44, *p* < 0.001). The subgroup analyses revealed a trend toward a superior yield for EBUS-TBMC in lymphoma (RD 0.11, *p* = 0.05) and sarcoidosis (RD 0.03, *p* = 0.077), while no significant differences were found for lung cancer subtypes. Safety profiles remained comparable, with no significant differences in the risk of pneumothorax (RD 0.00, *p* = 1.00) or bleeding (RD 0.00, *p* = 0.965). In conclusion, these findings support integrating EBUS-TBMC into diagnostic algorithms when preserved tissue architecture is critical, such as for lymphoproliferative disorders, granulomatous diseases, and advanced molecular profiling, providing a safe and more effective alternative to conventional needle aspiration.

## 1. Introduction

Endobronchial ultrasound-guided transbronchial needle aspiration (EBUS-TBNA) is a minimally invasive technique for sampling mediastinal lymph nodes, broadly used for staging lung cancer [1]. It has largely replaced mediastinoscopy because it is safer, less invasive, and provides comparable diagnostic accuracy. It is a technique that provides cytology samples rather than tissue specimens. This is one of its limits in diagnosing uncommon malignancies such as mediastinal lymphoma. However, it remains very convenient for non-small cell lung cancer (NSCLC) as the literature studies prove that it has a very low rate of inadequacy for mutation analysis [2]. A full battery of tests can be performed on these samples—immunocytochemistry, molecular testing, and even flow cytometry or microbiology in selected cases [3]. Next-generation sequencing (NGS) is feasible using these samples, and even whole-genome and whole-exome sequencing approaches have been evaluated for their effectiveness [4,5]. The most frequent complication of the procedure is minor bleeding, whereas other complications are rare (such as mediastinal infections, hypoxemia, pneumothorax, tracheobronchial perforation, vocal cord paralysis, and transient fever or vasovagal reactions) [6].

Cryotechnology has recently been used as a sampling tool in mediastinal and pulmonary pathology. The cryobiopsy probe benefits from the Joule–Thomson effect, which occurs when a compressed gas is released at high flow, expanding and significantly reducing the surrounding temperature [7]. Endobronchial ultrasound-guided transbronchial mediastinal cryobiopsy (EBUS-TBMC) is a method that targets the collection of tissue samples from mediastinal lymph nodes or masses. It uses a cryoprobe, inserted with ultrasonographic guidance through the EBUS needle site or, less frequently, through a small electrocautery incision [8]. It has an advantage over EBUS-TBNA—it obtains larger, structurally preserved tissue samples, free from crush artefacts [9]. There are studies in the literature that point out EBUS-TBMC superiority compared to EBUS-TBNA and endoscopic ultrasound-guided fine needle aspiration (EUS-FNA), as it is more accurate in lymphoma subtyping [10] and diagnosing benign disorders such as sarcoidosis [11]. TBMC is proven safe in comorbid patients and in an outpatient setting. The management of antithrombotic therapy is the same as EBUS TBNA [12].

This study aimed to compare the diagnostic performance of convex probe EBUS-TBNA and EBUS-TBMC in sampling mediastinal and hilar lymph nodes. We evaluate their diagnostic yields and the procedure’s safety. The objective was to determine whether cryobiopsy provides a significant diagnostic advantage over conventional needle aspiration and to assess its potential role in standard diagnostic practice in thoracic oncology and pulmonology.

## 2. Materials and Methods

### 2.1. Study Design and Protocol

This study was conducted as a systematic review and meta-analysis comparing the diagnostic yield, sample adequacy, and safety profile of EBUS-TBNA and EBUS-TBMC for mediastinal lesion sampling. It was reported in accordance with the Preferred Reporting Items for Systematic Reviews and Meta-Analyses (PRISMA 2020) guidelines.

The Population, Intervention, Comparison, and Outcome (PICO) model was applied to define the inclusion criteria for relevant studies. The population included adult patients undergoing endobronchial ultrasound procedures. The interventions of interest were EBUS-TBMC and EBUS-TBNA. The comparison group consisted of patients receiving EBUS-TBNA alone. The primary outcomes evaluated were diagnostic yield and procedure-related complications.

### 2.2. Information Sources and Search Strategy

A comprehensive literature search was performed from different databases by using PubMed, Web of Science, Google Scholar, ResearchGate and Embase by using the following words: ((((((((EBUS-TBNA) AND (endobronchial ultrasound transbronchial needle aspiration)) OR (EBUS TMC)) AND (endobronchial ultrasound transbronchial mediastinal cryobiopsy)) AND (mediastinal biopsy)) AND (diagnostic yield)) AND (mediastinal cryobiopsy)) OR (cryo-nodal biopsy)) OR (lymph node cryobiopsy)). The search covered publications from January 2020 to July 2025.

### 2.3. Eligibility Criteria

Studies were eligible if published between January 2020 and July 2025 and included adult human participants undergoing mediastinal sampling with EBUS-guided techniques. Eligible studies were required to provide either a direct comparison between EBUS-TBNA and EBUS-TBMC or sufficient extractable quantitative data for at least one of these procedures, and to report at least one of the outcomes of interest, namely diagnostic yield, sample adequacy, sample quality, and procedure-related complications.

Studies were excluded if they were review articles, editorials, letters, conference abstracts, posters, or non-peer-reviewed publications, as well as studies lacking sufficient extractable quantitative data or those not using standardised EBUS-guided sampling techniques. Studies focusing exclusively on non-mediastinal lesions or on unrelated pathologies were also excluded.

### 2.4. Study Selection Process

All retrieved records were exported into a reference management software, and the duplicates were removed. Two reviewers (L.S.M) and (D.M.V) independently screened the titles and abstracts to assess eligibility. Disagreements were solved by discussion. Then, the same authors assessed the full text of potentially relevant studies. Disagreements were resolved through discussion and consensus, with adjudication by a third reviewer when necessary. Studies fulfilling the eligibility criteria were included in both the qualitative and quantitative synthesis.

A total of 20 studies met the eligibility criteria and were included in the qualitative and quantitative synthesis.

### 2.5. Data Collection Process and Data Items

Data extraction was performed independently by two reviewers using a standardised data collection form. Extracted information included study characteristics (author, year, country, and study design), number of patients, biopsy technique used, target lymph node stations, diagnostic yield, sample adequacy, complication rates, and the type and severity of reported complications. Any discrepancies between reviewers were resolved through verification of the article text and discussion (D.C.L.).

### 2.6. Risk of Bias Assessment

We assessed the methodological quality and risk of bias of the included studies using the modified version of QUADAS-2 tool, tailored to comparative EBUS diagnostic yield studies (EBUS-TBNA vs. EBUS-TBMC) by the authors of the study (L.S.M., D.M.V., and D.C.L). Since there is no current agreed-upon standard test, we renamed the index and the standard tests to the first and second tests. The question regarding the appropriateness of the standard test was removed and replaced with question 2 from the index test, thus preserving the same methodological evaluation for both tests. The questions for both tests were ordered the same. From the flow and timing domain, the questions regarding the standard test were removed. The complete modified version is presented in the Supplementary Materials. Two reviewers independently judged the risk of bias and applicability concerns across four domains: patient selection, first test, second test, and flow and timing. The outcome was defined as the final diagnosis established by histopathology (samples obtained through TBNA or cryobiopsy). Each domain was rated as a low, high, or unclear risk of bias according to the prespecified signalling questions (Supplementary Materials). The rules for producing an overall risk of bias rating for each domain are as follows: If all signalling questions within the domain are answered ‘yes’, then the risk of bias for this domain is rated ‘low.’ [low risk]. If at least one signalling question within the domain is answered ‘no’, then the risk of bias for this domain is rated ‘high.’ [high risk]. If at least one signalling question within the domain is answered ‘unclear’ while the remaining signalling questions are answered ‘yes’, then the risk of bias is rated ‘unclear’ [unclear risk]. Disagreements were resolved by consensus and a discussion with the third reviewer (D.C.L.).

### 2.7. Statistical Analysis

A meta-analysis was performed to estimate the difference in percentages between the compared groups across all included studies using a random-effects model. Between-study heterogeneity was assessed with the I^2^ heterogeneity index, and the Cochran’s Q test for heterogeneity was calculated. A funnel plot was constructed to evaluate the potential publication bias, and a forest plot was generated to summarise the meta-analytic results. For all statistical tests, a significance level of 0.05 was used, and two-sided *p*-values were considered when available. Statistical analyses were conducted using the R statistical computing environment (R Foundation for Statistical Computing, Vienna, Austria) version 4.3.2, and the meta-analysis was performed using the meta package version 6.5.0.

## 3. Results

**Figure 1:**
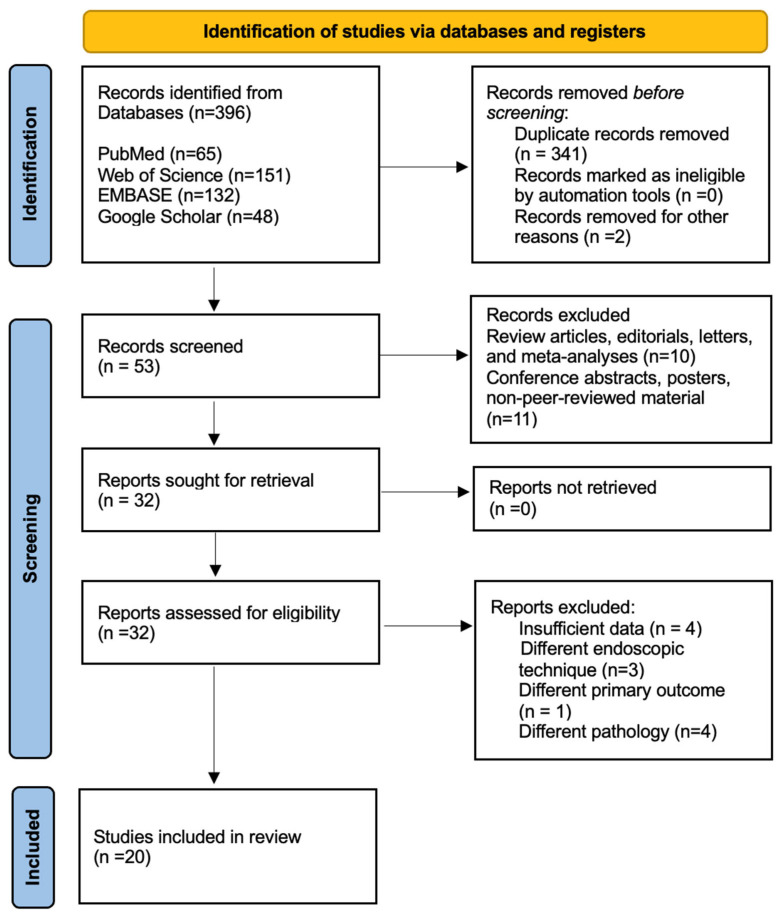
A flow chart illustrating the identification, selection, and inclusion of articles.

A total of 396 records were identified through systematic database searching across PubMed (n = 65), Web of Science (n = 151), EMBASE (n = 132), and Google Scholar (n = 48). After the removal of duplicates and ineligible entries, 53 studies were screened by their titles and abstracts. Of these, 32 articles were retrieved for full-text assessment. Following the application of the predefined inclusion and exclusion criteria, 20 studies were deemed eligible and included in the final qualitative and quantitative synthesis. The identification and selection process is illustrated in Figure 1.

The included studies were published between January 2020 and July 2025, involved adult human subjects, and provided comparative data on at least one of the outcomes of interest—diagnostic yield, sample adequacy, or complication rate—for both EBUS-TBNA and EBUS-TBMC. The studies varied in sample size, clinical indications, and methodology, but all met the minimum quality threshold for inclusion in meta-analysis.

### 3.1. Study Characteristics

The main characteristics of the included studies are presented in Table 1 and Table 2.

### 3.2. Diagnostic Efficacy

**Figure 2:**
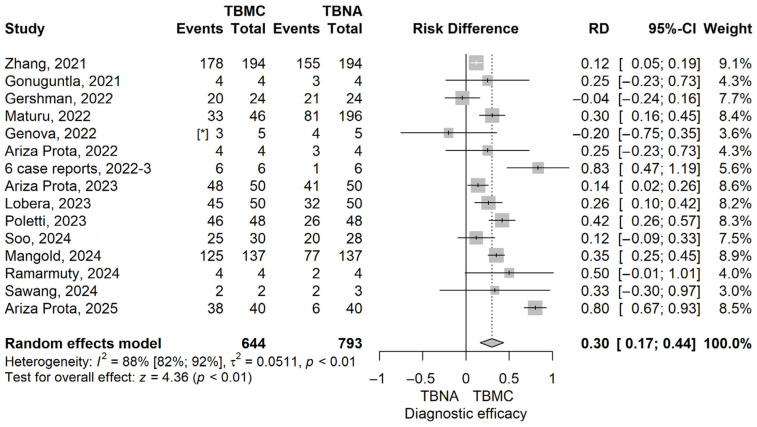
Forest plot for the diagnostic efficacy, comparing TBMC with TBNA [13,14,15,16,17,18,19,20,21,22,23,24,25,26]. Solid line represents the line of no effect. Dashed line represents the overall effect. Solid. *, 6 case reports combined [27,28,29,30,31,32]. RD, risk difference; CI, confidence interval; TBNA, transbronchial needle aspiration; TBMC, transbronchial mediastinal cryobiopsy.

The TBMC group had higher diagnostic efficacy compared to the TBNA group; the risk difference (RD) value obtained with the meta-analysis was 0.3 (95% CI 0.17–0.44), *p* ≤ 0.001 (Figure 2). The observed statistical heterogeneity was of 88.2% (95% CI 82.2–92.2%), *p* ≤ 0.001.

A leave-one-out sensitivity analysis was performed, and no matter which study was excluded, the final result remained statistically significant and in the same direction (Supplementary Materials, Figure S1). The heterogeneity for this analysis remained between 73% and 88%. The funnel plot for the diagnostic efficacy, comparing TBMC with TBNA, is shown in Figure S2 in the Supplementary Materials. The publication bias test gave a *p* = 0.585.

### 3.3. Safety Outcomes and Procedure Complications

Among the included studies, the main reported complications were bleeding and pneumothorax. Based on the pooled analysis, there were no statistically significant differences between the two techniques regarding the risk of these adverse events.

**Figure 3:**
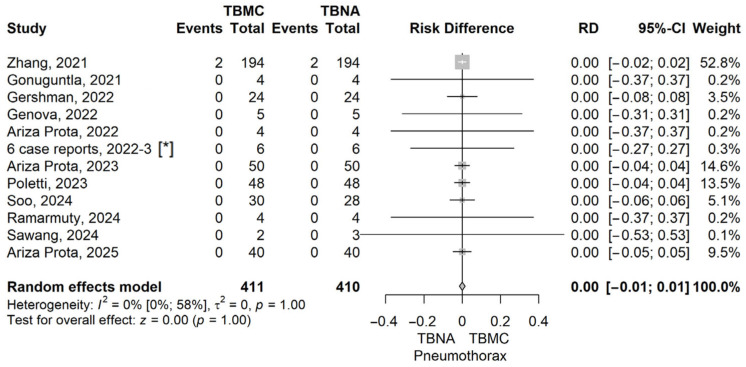
Forest plot for pneumothorax, comparing TBMC with TBNA [13,14,15,16,19,20,22,23,24,25,26]. Solid line represents the line of no effect. Dashed line represents the overall effect. *, 6 case reports combined [27,28,29,30,31,32]. RD, risk difference; CI, confidence interval; TBNA, transbronchial needle aspiration; TBMC, transbronchial mediastinal cryobiopsy.

The RD value (the RD of Pneumothorax in the TBMC group compared to the TBNA group) obtained with the meta-analysis was 0 (95% CI −0.01 to –0.01), *p* = 1, using the model with random effects. The heterogeneity was assessed, and we found an I^2^ of 0% (95% CI 0–58%), and the Q test for heterogeneity gave *p* = 1 (Figure 3). The funnel plot for Pneumothorax comparing TBMC with TBNA is shown in the Supplementary Materials, Figure S3.

**Figure 4:**
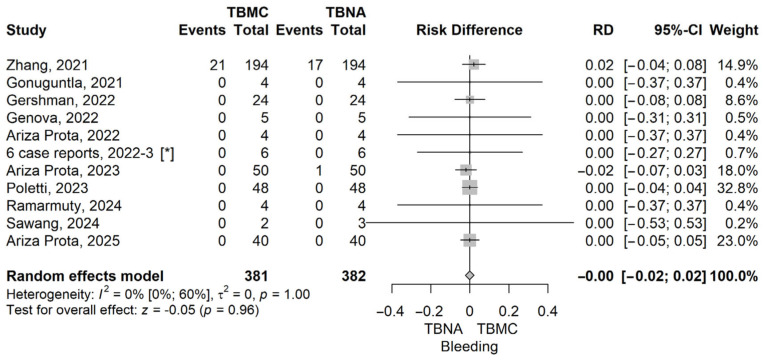
Forest plot for bleeding, comparing TBMC with TBNA [13,14,15,19,20,22,23,24,25,26]. Solid line represents the line of no effect. Dashed line represents the overall effect. *, 6 case reports combined [27,28,29,30,31,32]. RD, risk difference; CI, confidence interval; TBNA, transbronchial needle aspiration; TBMC, transbronchial mediastinal cryobiopsy.

The RD value (the RD of bleeding in the TBMC group compared to the TBNA group) obtained from the meta-analysis was 0 (95% CI: −0.02 to 0.02), *p* = 0.965, using a random-effects model. The heterogeneity was assessed, and we found an I^2^ of 0% (95% CI 0–60%), and the Q test for heterogeneity gave *p* = 1 (Figure 4). The funnel plot for bleeding, comparing TBMC with TBNA, is shown in Figure S4 from the Supplementary Materials.

### 3.4. Analysis per Pathology

**Figure 5:**
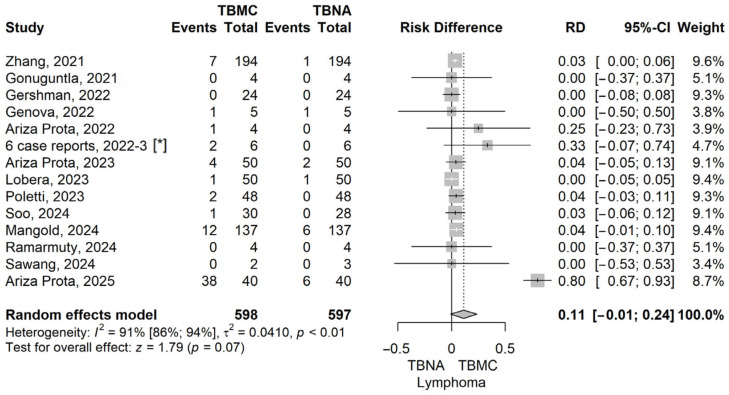
Forest plot for lymphoma, comparing TBMC with TBNA [13,14,15,16,17,18,19,20,22,23,24,25,26]. Solid line represents the line of no effect. Dashed line represents the overall effect. *, 6 case reports combined [27,28,29,30,31,32]. RD, risk difference; CI, confidence interval; TBNA, transbronchial needle aspiration; TBMC, transbronchial mediastinal cryobiopsy.

**Figure 6:**
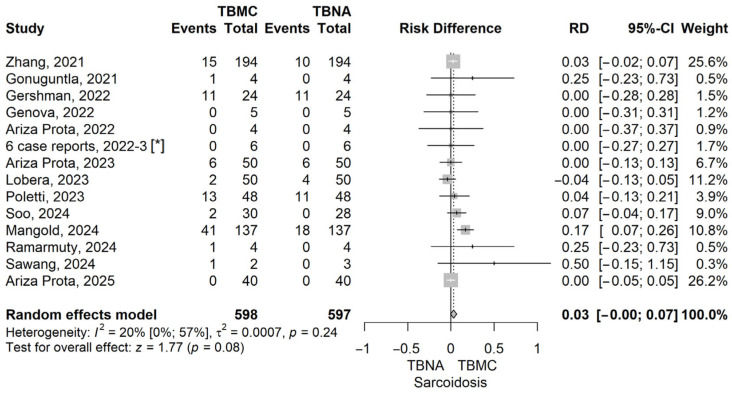
Forest plot for sarcoidosis, comparing TBMC with TBNA [13,14,15,16,17,18,19,20,22,23,24,25,26]. Solid line represents the line of no effect. Dashed line represents the overall effect. *, 6 case reports combined [27,28,29,30,31,32]. RD, risk difference; CI, confidence interval; TBNA, transbronchial needle aspiration; TBMC, transbronchial mediastinal cryobiopsy.

**Figure 7:**
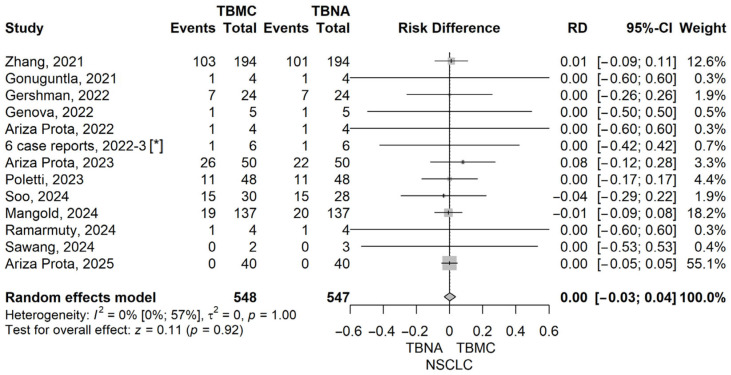
Forest plot for NSCLC, comparing TBMC with TBNA [13,14,15,16,17,19,20,22,23,24,25,26]. Solid line represents the line of no effect. Dashed line represents the overall effect. *, 6 case reports combined [27,28,29,30,31,32]. RD, risk difference; CI, confidence interval; TBNA, transbronchial needle aspiration; TBMC, transbronchial mediastinal cryobiopsy.

**Figure 8:**
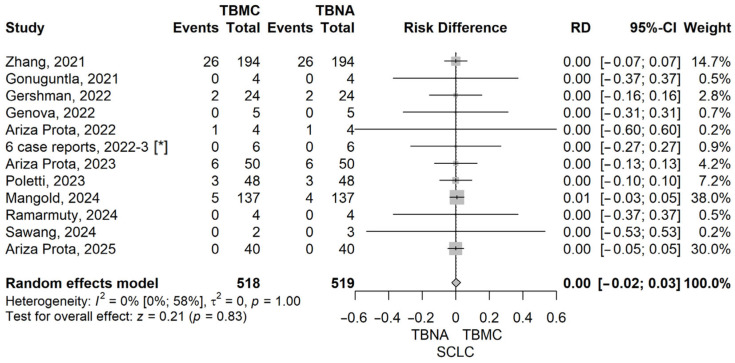
Forest plot for SCLC, comparing TBMC with TBNA [13,14,15,17,19,20,22,23,24,25,26]. Solid line represents the line of no effect. Dashed line represents the overall effect. *, 6 case reports combined [27,28,29,30,31,32]. RD, risk difference; CI, confidence interval; TBNA, transbronchial needle aspiration; TBMC, transbronchial mediastinal cryobiopsy.

To further explore the diagnostic performance of EBUS-TBMC versus EBUS-TBNA across specific clinical indications, we conducted analyses for patients with sarcoidosis, lymphoma, and lung cancer. These analyses aimed to identify whether EBUS-TBMC confers a measurable diagnostic advantage in distinct pathological settings, particularly those where preserved tissue architecture may play a critical role in establishing a definitive diagnosis. Forrest plots corresponding to each subgroup analysis are presented below (Figure 5, Figure 6, Figure 7 and Figure 8).

The pooled risk difference (RD) for the lymphoma subgroup was 0.11 (95% CI: –0.01 to 0.24), with a *p*-value of 0.05, using a random-effects model. Although the confidence interval includes the null value, the result demonstrates a trend toward statistical significance, suggesting that EBUS-TBMC may provide a modest diagnostic advantage over EBUS-TBNA in patients with suspected lymphoproliferative disorders (Figure 5).

The RD value for sarcoidosis, comparing TBMC to TBNA, was 0.03 (95% CI: 0 to 0.07), with a *p*-value of 0.077 under the random-effects model. Although not statistically significant, this result suggests a favourable trend toward improved diagnostic yield with EBUS-TBMC. The low heterogeneity (I^2^ = 20%) enhances the robustness of the estimate. Given the histological complexity of granulomatous diseases such as sarcoidosis, the ability of TBMC to retrieve larger, well-preserved tissue fragments may provide a clinical advantage in selected cases (Figure 6).

In the subgroup analyses for lung cancer, we evaluated the diagnostic performance separately for non-small cell lung cancer (NSCLC) and small cell lung cancer (SCLC). For NSCLC, the pooled risk difference (RD) between TBMC and TBNA was 0.00 (95% CI: −0.03 to 0.04, *p* = 0.915), and for SCLC, the RD was also 0.00 (95% CI: −0.02 to 0.03, *p* = 0.834), indicating no statistically significant difference in diagnostic yield for either histological subtype. Both analyses showed no heterogeneity (I^2^ = 0%), with non-significant Q tests (*p* = 1.0), supporting the consistency of the findings across the included studies. These results suggest that in lung cancer—where cytological material is often sufficient for diagnosis and molecular testing—the added value of TBMC over TBNA may be limited. Forest plots illustrating these findings are provided in Figure 7 and Figure 8.

### 3.5. Risk of Bias Assessment

The methodological quality of the included studies was assessed using the QUADAS-2 tool. Overall, the majority of studies showed a low risk of bias in the index test and flow and timing domains, reflecting the standardised conduct of EBUS-guided procedures and consistent reporting of patient flow across studies.

The patient selection domain presented the greatest variability, with several studies rated as high risk, mainly due to small case series designs, retrospective inclusion, or lack of clearly reported consecutive patient enrolment. It is unlikely that the studies that had unclear reporting of exclusion would have a high risk of bias. Thus, the most likely rating would be low in those cases. There were no case–control designs that interfered with the quality of the methodology. About half (57%) of the studies (8) were assessed as high bias, 14% (2) were assessed as unclear, and 29% (4) were assessed as low bias.

For the first and second test domains, the risk of bias was frequently rated as unclear, primarily because many studies did not explicitly report whether the final diagnostic adjudication was performed blinded to the other test results or did not provide sufficient detail regarding verification procedures. For the first test, 14% (2) were assessed as a high risk, 79% (11) were assessed as an unclear risk, and 7% (1) were assessed as a low risk. For the second test, 7% (1) were assessed as a high risk, and 93% (13) were assessed as an unclear risk. In reality, it is likely that many studies were unblinded; thus, the percentage of high-risk studies would be higher.

Concerning the flow and timing domain, all participants were included in the analyses, and the time between the tests was appropriate; thus, all the studies were at a low risk of bias.

Applicability concerns were generally low across all domains, as the study populations, index procedures (EBUS-TBNA and EBUS-TBMC), and diagnostic objectives were highly consistent with the review question. A summary of the risk-of-bias and applicability assessments is presented in Table 3.

## 4. Discussion

Our meta-analysis demonstrated a statistically significant superiority of EBUS-TBMC over EBUS-TBNA in terms of diagnostic efficacy, with a pooled risk difference (RD) of 0.30 [95% CI: 0.17–0.44], *p* < 0.001. This translates into an absolute increase of 30% in diagnostic yield for TBMC, highlighting its added value across a range of intrathoracic pathologies. Despite the high between-study heterogeneity (I^2^ = 88%), sensitivity analyses confirmed the robustness of this effect, with no individual study significantly altering the overall result.

These findings are consistent with the results of Mathew et al. (2024), who reported a diagnostic yield of 91% for TBMC and 81% for TBNA (OR = 2.5; 95% CI: 1.6–3.91), based on a separate meta-analysis [11]. Their subgroup analysis demonstrated a particularly strong benefit for TBMC in benign diseases and lymphoproliferative disorders, with odds ratios of 7.95 and 11.48, respectively—further supporting the importance of preserved tissue architecture in these contexts. Moreover, our conclusions align with those of Romero and Kho (2024), who emphasised in their literature review that EBUS-TBMC offers superior diagnostic performance, especially in cases requiring an intact histological structure, such as lymphomas and granulomatous disease [33]. Although their review did not include a pooled quantitative estimate, it corroborated the diagnostic value and safety profile of EBUS-TBMC.

Despite its diagnostic advantages, EBUS-TBMC presents several limitations. Soo et al. (2025) reported bleeding as the most common complication, and other studies highlight increased procedural complexity, higher costs, and a steeper learning curve [34]. Crucially, while procedure-related bleeding is frequently reported, it is important to distinguish between minor intraprocedural bleeding and clinically significant bleeding. In our analysis, the lack of difference in bleeding risk between the two procedures stems from the use of standardised prophylactic methodologies in the protocols of the included studies and the low incidence of major bleeding requiring intervention in both groups. Furthermore, EBUS-TBMC has a relatively low incidence of pneumothorax and pneumomediastinum, reported in approximately 1% of cases, events that are typically self-limiting and do not require intervention [33].

Emerging studies suggest that EBUS-TBMC may overcome several limitations of conventional needle aspiration, particularly by providing larger, better-preserved tissue samples suitable for histological and molecular analyses [35]. Its diagnostic value has been increasingly recognized in conditions where tissue architecture is critical. 

Regarding the NSCLC subgroup, our meta-analysis demonstrated a pooled risk difference (RD) of 0.00 (95% CI: −0.03 to 0.04, *p* = 0.915), indicating no significant diagnostic advantage for either EBUS-TBMC or EBUS-TBNA. These findings are characterized by exceptional robustness, as evidenced by the negligible heterogeneity (I^2^ = 0%, *p* = 1.00). This result is highly consistent with the landmark randomized controlled trial conducted by Zhang et al. (2021), which similarly reported no statistically significant difference in diagnostic yield for common lung cancer between TBNA and cryobiopsy (94.1% vs. 95.6%, *p* = 0.58) [15]. The alignment between our pooled evidence and Zhang’s trial data reinforces the conclusion that for the primary diagnosis of NSCLC, both techniques achieve near-optimal performance.

In the lymphoma subgroup, our meta-analysis revealed a modest but favourable diagnostic advantage for EBUS-TBMC over EBUS-TBNA (RD = 0.11 [95% CI: –0.01 to 0.24], *p* = 0.05), although this did not reach statistical significance. This result should be interpreted in the context of substantial heterogeneity (I^2^ = 91%), likely due to clinical and methodological variation across studies. Notably, one of the largest studies included in our analysis, conducted by Ye Fan et al. (2023), demonstrated a significantly higher diagnostic yield for TBMC compared to TBNA in patients with lymphoma (91.3% vs. 47.8%, *p* = 0.019) [36]. While not an independent comparison, their findings exemplify the observed trend in favour of TBMC and underscore its potential diagnostic utility in lymphoproliferative disorders where preserved tissue architecture is essential for accurate subtyping.

In the sarcoidosis subgroup, our meta-analysis showed a small, non-significant trend favouring TBMC over TBNA (RD = 0.03 [95% CI: –0.00 to 0.07], *p* = 0.08), with low heterogeneity (I^2^ = 20%). These results are in line with those reported in the meta-analysis by Zhang et al. (2024), which included 15 studies and 1101 patients. Their pooled analysis showed a higher diagnostic yield for TBMC in sarcoidosis (90.0%) compared to TBNA (79.2%), with a statistically significant difference (*p* < 0.05) [37]. Although our own results did not reach significance, both meta-analyses support the notion that cryobiopsy may improve diagnostic accuracy in granulomatous conditions by providing larger, more intact tissue specimens. Also, these findings align with the larger diagnostic advantage reported in the randomised controlled trial by Deng et al., where TBMC achieved a significantly higher granuloma detection rate than TBNA (89.8% vs. 78.1%, *p* < 0.001) and a superior histological quality [38].

It must be acknowledged that while EBUS-guided techniques, particularly TBMC, significantly enhance tissue yield compared to conventional needle aspiration, the total volume of tissue obtained remains inferior to that of surgical mediastinoscopy performed under direct visualisation. However, the value of these minimally invasive approaches is most evident in clinical scenarios where surgical re-intervention is required. In cases of ‘redo’ mediastinoscopy, where the surgical risk is substantially increased due to post-operative adhesions and altered anatomy, EBUS-TBNA and TBMC provide a safer and highly effective diagnostic alternative [39].

## 5. Strengths and Limitations

This study has several strengths, including adherence to PRISMA 2020 methodological standards, a comprehensive and up-to-date literature search across five major databases, and the inclusion of recently published prospective studies. In addition, the analyses stratified by major pathological entities provided clinically relevant insights into the specific scenarios in which EBUS-TBMC may offer the greatest diagnostic benefit.

Importantly, this meta-analysis has direct clinical applicability, as it provides quantitative evidence supporting the integration of EBUS-TBMC into diagnostic algorithms, particularly in conditions where preserved tissue architecture is essential, such as lymphoproliferative disorders and granulomatous diseases. By demonstrating a significant improvement in diagnostic yield without compromising safety, our findings may contribute to reducing the need for more invasive procedures, such as mediastinoscopy, and to optimising patient management in real-world clinical practice.

Furthermore, the simultaneous evaluation of diagnostic performance and safety outcomes enhances the translational value of the results, supporting their implementation in routine pulmonology and thoracic oncology practice.

This meta-analysis has several limitations that must be acknowledged. First, there was substantial heterogeneity among the included studies (I^2^ = 88%), likely reflecting differences in study design, patient populations, operator experience, procedural protocols, and criteria for diagnostic yield. Second, many studies had relatively small sample sizes and were conducted in single centres, which may limit the generalizability of the findings. Additionally, publication bias cannot be completely excluded, although a funnel plot analysis and Egger’s test did not show significant asymmetry. Several studies presented a high risk of bias due to small case series designs, retrospective inclusion, or unclear reporting of consecutive patient enrolment. For the two test domains, the risk of bias was frequently classified as unclear because many studies did not report whether diagnostic assessments were performed blinded.

## 6. Conclusions

This meta-analysis supports integrating EBUS-TBMC as a complementary or alternative approach to EBUS-TBNA, particularly when high-quality tissue architecture is essential. Its ability to provide larger, well-preserved specimens is critical for accurate lymphoma subtyping, diagnosing granulomatous diseases like sarcoidosis, and advanced molecular profiling in NSCLC. By enhancing histological quality, EBUS-TBMC may reduce diagnostic delays and the necessity for more invasive surgical procedures. Regarding safety, EBUS-TBMC has a complication rate similar to conventional EBUS-TBNA. Although cryobiopsy carries a slightly higher risk of bleeding, it can be safely managed using standardised protocols. However, a widespread clinical adoption requires further validation through large-scale, multicentre randomised controlled trials to establish standardised protocols. Future research should also address cost-effectiveness, training curves, and longitudinal outcomes to determine the long-term impact of EBUS-TBMC on patient management and prognostic stratification in pulmonology practice.

## Figures and Tables

**Table 1:** The main characteristics of the included studies.

No.	Authors	Study Design No. of Participants Country	The Type of Procedure	Diagnosis	Diagnostic Yield (%)	Complications (%)	Conclusion/Particularities
1.	Gershman et al., 2022 [13]	Prospective study from Israel n = 24	EBUS-TBNA	All histologies (lung cancer, metastatic carcinoma, and benign lesions)	87.5%	No complications	The study results showed a higher diagnostic yield for EBUS-TBNA than for cryobiopsy.
			EBUS-TBMC	All histologies (lung cancer, metastatic carcinoma, and benign lesions)	83.3%	No complications	
2.	Ariza Prota et al., 2023 [14]	Prospective study from Israel n = 50	EBUS-TBNA	All histologies (lung cancer, lymphoma, and benign conditions)	82%	Bleeding 18% Pneumothorax 0% Pneumomediastinum 0%	EBUS-TBMC appears to provide a superior overall diagnostic yield compared to EBUS-TBNA, particularly in identifying lymphoma, tumours of extrapulmonary origin, and lung carcinomas requiring molecular analysis.
			EBUS-TBMC	All histologies (lung cancer, lymphoma, and benign conditions)	96%	Bleeding 3.7% Pneumothorax 0% Pneumomediastinum 0%	
3.	Jing Zhang et al., 2021 [15]	Randomised clinical trial. 1:1 randomisation EBUS TBNA first vs. EBUS TBMC first at two hospital sites in Europe and Asia n = 197	EBUS-TBNA+ EBUS-TBMC (EBUS TBNA first)	All histologies (lung cancer, metastatic carcinoma, benign disorders, and uncommon tumours)	79.9%	Bleeding 84.7% Pneumothorax 1% Pneumomediastinum 0%	The diagnostic yield of mediastinal cryobiopsy is significantly higher than that of TBNA biopsies for both uncommon tumours and benign lesions; furthermore, cryobiopsy samples allow for more detailed pathological information through immunohistochemistry staining that is not achievable with corresponding TBNA samples.
			EBUS-TBNA+ EBUS-TBMC (EBUS TBMC first)	All histologies (lung cancer, metastatic carcinoma, benign disorders, and uncommon tumours)	91.8%	Bleeding 87.8% Pneumothorax 1% Pneumomediastinum 1%	
4.	Soo et al., 2024 [16]	Retrospective study from Malaysia n = 30	EBUS TBNA	All histologies (lung cancer and others)	71.4%	Bleeding-not assessed Pneumothorax 0% Pneumomediastinum 0%	EBUS-TBMC recorded an overall non-significantly higher diagnostic yield when compared to EBUS-TBNA. A more important clinical implication lies in the ability of EBUS-TBMC to provide sufficient tissue for complete molecular profiling in the treatment of NSCLC.
			EBUS TBMC	All histologies (lung cancer and others)	83.3%	Bleeding 16.7% Pneumothorax 0% Pneumomediastinum 0%	
5.	Mangold et al., 2024 [17]	Prospective study from Switzerland n = 137	EBUS TBNA	All histologies (lung cancer, other metastatic carcinoma, benign disorders, and uncommon tumours)	56.2%	Not individually assessed for each procedure	EBUS-guided cryobiopsy offers a significantly higher diagnostic yield for mediastinal and hilar conditions compared to EBUS-TBNA, particularly for benign diseases and uncommon tumours, as its larger sample size is better suited for immunohistochemistry of various tumour markers while maintaining a favourable safety profile.
			EBUS TBMC	All histologies (lung cancer, other metastatic carcinoma, benign lesions, and uncommon tumours)	91.2%	Not individually assessed for each procedure	
			EBUS TBNA + TBMC	All histologies (lung cancer, other metastatic carcinoma, benign lesions, and uncommon tumours)	82.35%	No complications	
6.	Lobera et al., 2023 [18]	Prospective study from Spain n = 50	EBUS-TBNA	All histologies (malignancies and benign lesions)	64%	Not assessed	The use of EBUS-TBCB significantly improves the diagnostic yield compared to EBUS-TBNA, with a low number of procedure-related complications. It is efficient, especially in benign pathologies that require high-quality histological samples.
			EBUS-TBMC	All histologies (malignancies and benign lesions)	90%	Bleeding 6% Pneumothorax 0% Pneumomediastinum 0%	
7.	Gonuguntla et al., 2021 [19]	Case series from India n = 4	EBUS-TBNA	All histologies (lung cancer and benign lesions)	75%	Not assessed	EBUS-TBMC was superior to EBUS-TBNA for the specific diagnosis of benign pathologies (sarcoidosis and tuberculosis).
			EBUS-TBMC	All histologies (lung cancer and benign lesions)	100%	Bleeding 25% Pneumothorax 0% Pneumomediastinum 0%	
8.	Poletti et al., 2023 [20]	Retrospective study from Italy n = 48	EBUS-TBNA	All histologies (lung cancer Hodgkin lymphoma, benign lesions, and rare mediastinal tumour—hamartoma, mesothelioma, pulmonary artery sarcoma, lung carcinoid, Castleman disease, and silicosis)	54.1%	No complications	EBUS-TMC may contribute to the precise diagnosis and subtyping of mediastinal diseases, especially lymphomas and rare mediastinal tumours, thereby reducing the number of nondiagnostic procedures.
			EBUS-TBMC	All histologies (lung cancer Hodgkin Lymphoma, benign lesions, and rare mediastinal tumour—hamartoma, mesothelioma, pulmonary artery sarcoma, lung carcinoid, Castleman disease, and silicosis)	95.8%	No complications	
9.	Maturu et al., 2022 [21]	Prospective study from India n = 196	EBUS-TBNA	All histologies	41.3%	Not assessed	The tissue obtained by EBUS-MCB is adequate for ancillary molecular and microbiologic studies. EBUS-MCB also has an acceptable safety profile.
			EBUS-TBMC	All histologies	71.7%	Bleeding 30%	
10.	Ariza Prota et al., 2025 [22]	Retrospective observational study 2025 n = 40	EBUS-TBNA	Lymphoma	15%	No complications	The overall sensitivity of EBUS-TMC was significantly higher compared to EBUS-TBNA alone and EBUS-TBNA + flow cytometry. This technique reduces the need for procedural repetitions and avoids more invasive and costly interventions such as mediastinoscopy.
			EBUS-TBMC	Lymphoma	95%		
11.	Genova et al., 2022 [23]	Case series from Italy n = 5	EBUS-TBNA	All histologies (lung cancer, lymphoma, and sarcoidosis)	60%	Not assessed	Although there was a difference in diagnostic yield, EBUS-TBNA still detected lymphoma cells, while cryobiopsy provided a more precise characterisation, revealing the presence of diffuse large B-cell lymphoma.
			EBUS-TBMC	All histologies (lung cancer, lymphoma, and sarcoidosis)	40%	Not assessed	
12.	Ariza Prota et al., 2022 [24]	Case series from Spain n = 4	EBUS-TBNA	Lung cancer	75%	No complications	EBUS-TMC could complement existing diagnostic methods for mediastinal diseases, particularly in cases involving rare tumours, suspected lymphoproliferative disorders, or the need for larger biopsy samples for molecular analysis.
			EBUS-TBMC	Lung cancer lymphoma	100%	No complications	
13.	Sawang et al., 2024 [25]	n = 3	EBUS-TBNA	Benign lesions	66.66%	No complications	EBUS-MCB is a useful method to provide a minimally invasive evaluation in addition to EBUS-TBNA and ROSE in patients with enlarged mediastinal nodes, with minimal complications in less time. Also, this can help avoid further investigations, such as mediastinoscopy.
			EBUS-TBMC	Benign lesions	100%	No complications	
14.	Ramarmuty, 2024 [26]	n = 4	EBUS-TBNA	Lung cancer and breast cancer Benign lesions (non-diagnostic)	100%	No complications	EBUS-TBMC was superior to EBUS-TBNA for the specific diagnosis of benign pathologies (silicosis and tuberculosis).
			EBUS-TBMC	Lung cancer, breast cancer, and benign lesions	100%	No complications	

**Table 2:** The main characteristics of the included studies (case studies).

No.	Author, Year	The Type of Procedure	Diagnosis	Diagnostic Yield (%)	Complications (%)	Conclusion/Particularities
1	Takemura et al., 2023 [27]	EBUS-TBNA	Non diagnostic	0%	No complication	EBUS-cryobiopsy may provide additional diagnostic value to EBUS-TBNA for the histological and molecular evaluation of mediastinal and hilar lesions.
		EBUS-TBMC	SMARCA 4 Undifferentiated Tumour	100%		
2	Hetzel et al., 2023 [28]	EBUS-TBNA	NSCLC	100%	No complication	EBUS transbronchial lymph node cryobiopsy may contribute to the optimised molecular workup of NSCLC, with important information for patients’ treatment.
		EBUS-TBMC	NSCLC	100%		
3	Zhang et al., 2022 [29]	EBUS-TBNA	Non diagnostic	0%	No complication	Non-cautery assisted transbronchial mediastinal cryobiopsy might be a new sampling strategy for mediastinal diseases.
		EBUS-TBMC	Lymphoma	100%		
4	Sze Shyang Kho et al., 2022 [30]	EBUS-TBNA	Non diagnostic	0%	No complication	Diagnosis of tuberculous mediastinal and hilar lymphadenitis via EBUS/transbronchial cryobiopsy has been reported in the literature, and its role is further strengthened by demonstrating its superiority among three different biopsy modalities.
		EBUS-TBMC	Tuberculous lymphadenitis	100%		
5	Tamburrini et al., 2022 [31]	EBUS-TBNA	Non diagnostic	0%	No complication	The study suggests the potential use of EBUS-guided cryobiopsy for the assessment of mediastinal lymph nodes, especially where a larger tissue size is needed for testing.
		EBUS-TBMC	Lymphoma	100%		
6	Zhang et al., 2023 [32]	EBUS-TBNA	Non diagnostic	0%	No complication	Additional mediastinal cryobiopsy might bring extra benefits to the diagnosis and treatment of uncommon thoracic tumours.
		EBUS-TBMC	SMARCA 4 Undifferentiated Tumour	100%		

**Table 3:** The risk of bias assessment and applicability concerns of the included studies (modified QUADAS-2).

	Author, year		Gershman et al., 2022 [13]	Ariza Prota et al., 2023 [14]	Jing Zhang et al., 2021 [15]	Soo et al., 2024 [16]	Mangold et al., 2024 [17]	Lobera et al., 2023 [18]	Gonuguntla et al., 2021 [19]	Poletti et al., 2023 [20]	Maturu et al., 2022 [21]	Ariza Prota et al., 2025 [22]	Genova et al., 2022 [23]	Ariza Prota et al., 2022 [24]	Sawang et al., 2024 [25]	Ramarmuty et al., 2024 [26]
RISK OF BIAS	Patient Selection	Q1	Yes	No	Yes	No	Yes	Yes	No	No	Yes	No	No	Yes	No	No
		Q2	Yes	Yes	Yes	Yes	Yes	Yes	Yes	Yes	Yes	Yes	Yes	Yes	Yes	Yes
		Q3	Unclear	No	Yes	Unclear	Yes	Yes	No	Unclear	Yes	No	No	No	No	No
	First Test	Q1	Unclear	Unclear	Yes	Unclear	No	Unclear	Unclear	Unclear	Unclear	No	Unclear	Unclear	Unclear	Unclear
		Q2	Yes	Yes	Yes	Yes	Yes	Yes	Yes	Yes	Yes	Yes	Yes	Yes	Yes	Yes
	Second Test	Q1	Unclear	Unclear	Unclear	Unclear	Unclear	Unclear	Unclear	Unclear	Unclear	No	Unclear	Unclear	Unclear	Unclear
		Q2	Yes	Yes	Yes	Yes	Yes	Yes	Yes	Yes	Yes	Yes	Yes	Yes	Yes	Yes
	Flow and Timing	Q1	Yes	Yes	Yes	Yes	Yes	Yes	Yes	Yes	Yes	Yes	Yes	Yes	Yes	Yes
		Q2	Yes	Yes	Yes	Yes	Yes	Yes	Yes	Yes	Yes	Yes	Yes	Yes	Yes	Yes
	APPLICABILITY CONCERNS	Patient Selection	Low	High	Low	Low	Low	Low	High	Low	Low	Low	High	High	High	High
		Index Test	Low	Low	Low	Low	Low	Low	Low	Low	Low	Low	Low	Low	Low	Low
		Reference Standard	Low	Low	Low	Low	Unclear	Low	Low	Unclear	Low	Low	Low	Low	Low	Low

## Data Availability

The data supporting the findings of this study are available within the article and its Supplementary Materials.

## Supplementary Materials

The following supporting information can be downloaded at https://www.mdpi.com/article/10.3390/life16050768/s1. Supplementary Table S1: Prisma 2020 Checklist; Supplementary Figure S1: Leave-one-out sensitivity analysis plot for selected studies for Diagnostic efficacy; Supplementary Figure S2: Funnel plot for Diagnostic efficacy, comparing TBMC with TBNA; Supplementary Figure S3: Funnel plot for Pneumothorax, comparing TBMC with TBNA; Supplementary Figure S4: Funnel plot for Bleeding, comparing TBMC with TBNA; Supplementary Figure S5: Funnel plot for Lymphoma, comparing TBMC with TBNA; Supplementary Figure S6: Funnel plot for Sarcoidosis, comparing TBMC with TBNA; Supplementary Figure S7: Forrest plot for Non-small cell lung cancer, comparing TBMC with TBNA; Supplementary Figure S8: Forrest plot Small cell lung cancer, comparing TBMC with TBNA.

## Abbreviations

The following abbreviations are used in this manuscript:

EUS-FNA	Endoscopic Ultrasound-Guided Fine Needle Aspiration
EBUS-TBNA	Endobronchial Ultrasound-Guided Transbronchial Needle Aspiration
EBUS-TBMC	Endobronchial Ultrasound-Guided Transbronchial Mediastinal Cryobiopsy
EBUS	Endobronchial Ultrasound
TBNA	Transbronchial Needle Aspiration
TBMC	Transbronchial Mediastinal Cryobiopsy
NSCLC	Non-Small Cell Lung Cancer
SCLC	Small Cell Lung Cancer
RD	Risk Difference
CI	Confidence Interval
PRISMA	Preferred Reporting Items for Systematic Reviews and Meta-Analyses
PICO	Population, Intervention, Comparison, Outcome
QUADAS-2	Quality Assessment of Diagnostic Accuracy Studies 2
NGS	Next-Generation Sequencing
I2	I-squared (measure of heterogeneity)
