# Supplementary material for: Comparative Efficacy of Transbronchial Needle Aspiration and Cryobiopsies in Thoracic Disorders: A Systematic Review and Meta-Analysis for Optimal Diagnostic Efficacy
Source: Life (Basel). 2026 May 3;16(5):768. doi: 10.3390/life16050768 (PMC13208292; doi:10.3390/life16050768)
Supplement: Supplementary file 1 [file life-16-00768-s001.zip › life-4235349-supplementary.pdf]

Table S1.Prisma 2020 Checklist

Section and Topic	Item #	Checklist item	Location where item is reported
**TITLE**			
Title	1	Identify the report as a systematic review.	Page 1, Title
**ABSTRACT**			Page 1, Abstract
Abstract	2	See the PRISMA 2020 for Abstracts checklist.	
**INTRODUCTION**			
Rationale	3	Describe the rationale for the review in the context of existing knowledge.	Page 2, Introduction
Objectives	4	Provide an explicit statement of the objective(s) or question(s) the review addresses.	Page 2 Introduction (last paragraph)
**METHODS**			
Eligibility criteria	5	Specify the inclusion and exclusion criteria for the review and how studies were grouped for the syntheses.	Page 3, Section 2.3
Information sources	6	Specify all databases, registers, websites, organisations, reference lists and other sources searched or consulted to identify studies. Specify the date when each source was last searched or consulted.	Page 3, Section 2.2
Search strategy	7	Present the full search strategies for all databases, registers and websites, including any filters and limits used.	Page 3, Section 2.2
Selection process	8	Specify the methods used to decide whether a study met the inclusion criteria of the review, including how many reviewers screened each record and each report retrieved, whether they worked independently, and if applicable, details of automation tools used in the process.	Page 3, Section 2.4
Data collection process	9	Specify the methods used to collect data from reports, including how many reviewers collected data from each report, whether they worked independently, any processes for obtaining or confirming data from study investigators, and if applicable, details of automation tools used in the process.	Page 3, Section 2.5
Data items	10a	List and define all outcomes for which data were sought. Specify whether all results that were compatible with each outcome domain in each study were sought (e.g. for all measures, time points, analyses), and if not, the methods used to decide which results to collect.	Page 3, Section 2.5
	10b	List and define all other variables for which data were sought (e.g. participant and intervention characteristics, funding sources). Describe any assumptions made about any missing or unclear information.	Page 3, Section 2.5
Study risk of bias assessment	11	Specify the methods used to assess risk of bias in the included studies, including details of the tool(s) used, how many reviewers assessed each study and whether they worked independently, and if applicable, details of automation tools used in the process.	Page 3, Section 2.6
Effect measures	12	Specify for each outcome the effect measure(s) (e.g. risk ratio, mean difference) used in the synthesis or presentation of results.	Page 4, Section 2.7

Table S1.Prisma 2020 Checklist

Section and Topic	Item #	Checklist item	Location where item is reported
Synthesis methods	13a	Describe the processes used to decide which studies were eligible for each synthesis (e.g. tabulating the study intervention characteristics and comparing against the planned groups for each synthesis (item #5)).	Page 4, Section 2.7
	13b	Describe any methods required to prepare the data for presentation or synthesis, such as handling of missing summary statistics, or data conversions.	Page 4, Section 2.7
	13c	Describe any methods used to tabulate or visually display results of individual studies and syntheses.	Page 4, Section 2.7
	13d	Describe any methods used to synthesize results and provide a rationale for the choice(s). If meta-analysis was performed, describe the model(s), method(s) to identify the presence and extent of statistical heterogeneity, and software package(s) used.	Page 4, Section 2.7
	13e	Describe any methods used to explore possible causes of heterogeneity among study results (e.g. subgroup analysis, meta-regression).	Page 4, Section 2.7
	13f	Describe any sensitivity analyses conducted to assess robustness of the synthesized results.	Page 4, Section 2.7
Reporting bias assessment	14	Describe any methods used to assess risk of bias due to missing results in a synthesis (arising from reporting biases).	Page 3, Section 2.6
Certainty assessment	15	Describe any methods used to assess certainty (or confidence) in the body of evidence for an outcome.	Page 3, Section 2.6
**RESULTS**			
Study selection	16a	Describe the results of the search and selection process, from the number of records identified in the search to the number of studies included in the review, ideally using a flow diagram.	Page 5, Section Results, Figure 1
	16b	Cite studies that might appear to meet the inclusion criteria, but which were excluded, and explain why they were excluded.	Page 5, Section Results, Figure 1
Study characteristics	17	Cite each included study and present its characteristics.	Page 6-10 Tables 1-2
Risk of bias in studies	18	Present assessments of risk of bias for each included study.	Page 15, Section 3.5, Table 3
Results of individual studies	19	For all outcomes, present, for each study: (a) summary statistics for each group (where appropriate) and (b) an effect estimate and its precision (e.g. confidence/credible interval), ideally using structured tables or plots.	Page 6-10 Tables 1-2

Table S1.Prisma 2020 Checklist

Section and Topic	Item #	Checklist item	Location where item is reported
Results of syntheses	20a	For each synthesis, briefly summarise the characteristics and risk of bias among contributing studies.	Page 5-12, Sections 3.2, 3.3, and 3.4
	20b	Present results of all statistical syntheses conducted. If meta-analysis was done, present for each the summary estimate and its precision (e.g. confidence/credible interval) and measures of statistical heterogeneity. If comparing groups, describe the direction of the effect.	Page 5-12, Sections 3.2, 3.3, and 3.4
	20c	Present results of all investigations of possible causes of heterogeneity among study results.	Page 5-12, Sections 3.2, 3.3, and 3.4
	20d	Present results of all sensitivity analyses conducted to assess the robustness of the synthesized results.	Page 5-12, Sections 3.2, 3.3, and 3.4
Reporting biases	21	Present assessments of risk of bias due to missing results (arising from reporting biases) for each synthesis assessed.	Page 5-12, Sections 3.2, 3.3,
Certainty of evidence	22	Present assessments of certainty (or confidence) in the body of evidence for each outcome assessed.	Page 14, Section 3.5
**DISCUSSION**			
Discussion	23a	Provide a general interpretation of the results in the context of other evidence.	Page 15, Section 4
	23b	Discuss any limitations of the evidence included in the review.	Page 17, Section 5
	23c	Discuss any limitations of the review processes used.	Page 17, Section 5
	23d	Discuss implications of the results for practice, policy, and future research.	Page 17, Section 5
**OTHER INFORMATION**			
Registration and protocol	24a	Provide registration information for the review, including register name and registration number, or state that the review was not registered.	The review was not registered
	24b	Indicate where the review protocol can be accessed, or state that a protocol was not prepared.	The review was not registered

Table S1.Prisma 2020 Checklist

Section and Topic	Item #	Checklist item	Location where item is reported
	24c	Describe and explain any amendments to information provided at registration or in the protocol.	The review was not registered
Support	25	Describe sources of financial or non-financial support for the review, and the role of the funders or sponsors in the review.	No funding
Competing interests	26	Declare any competing interests of review authors.	No conflict of interests
Availability of data, code and other materials	27	Report which of the following are publicly available and where they can be found: template data collection forms; data extracted from included studies; data used for all analyses; analytic code; any other materials used in the review.	Supplementary file

SUPPLEMENTARY FILE

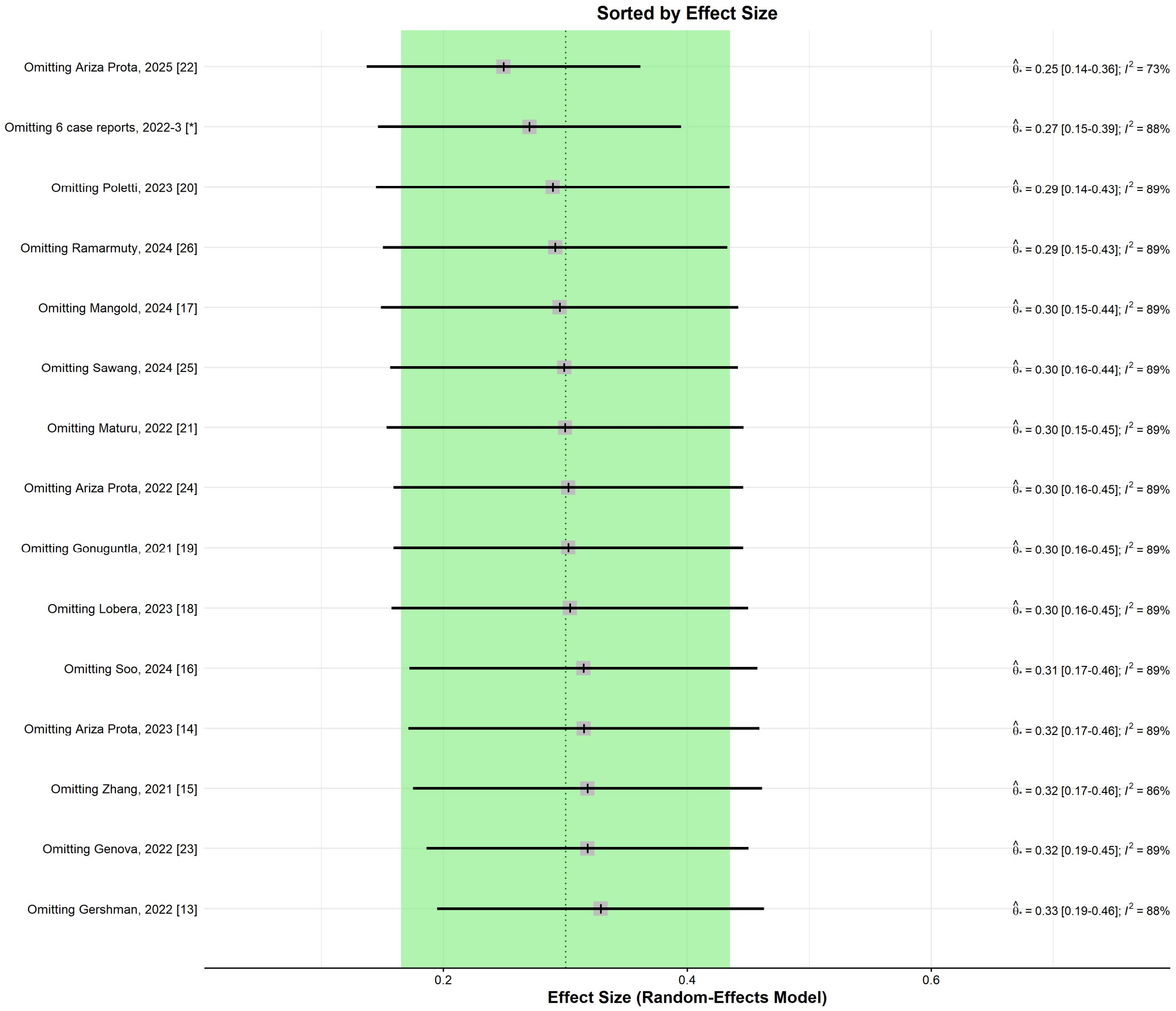

Fig S1. Leave-one-out sensitivity analysis plot for selected studies for Diagnostic efficacy

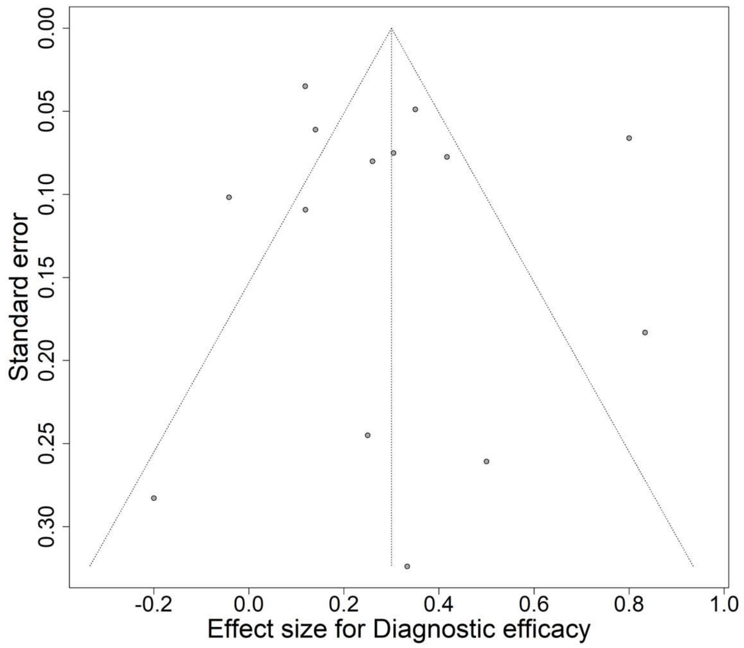

Fig.S2 Funnel plot for Diagnostic efficacy, comparing TBMC with TBNA

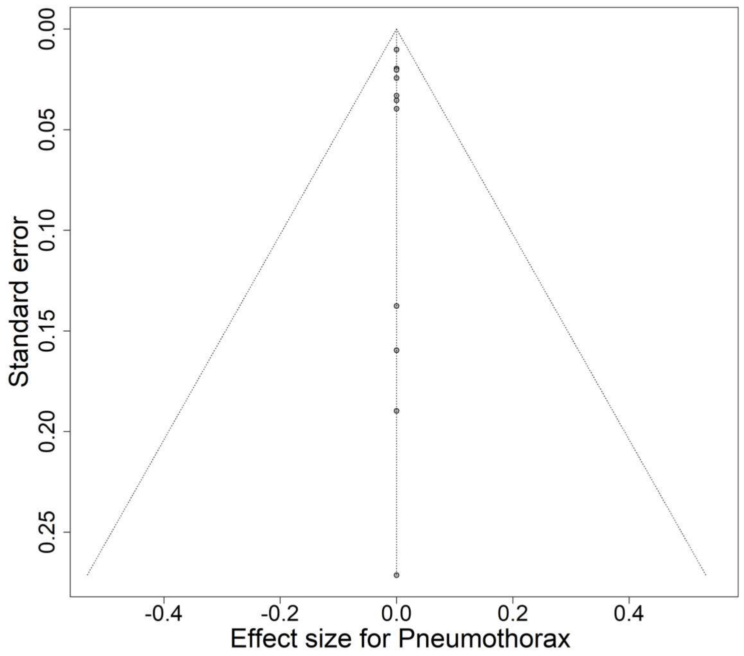

Fig S3. The funnel plot for Pneumothorax, comparing TBMC with TBNA

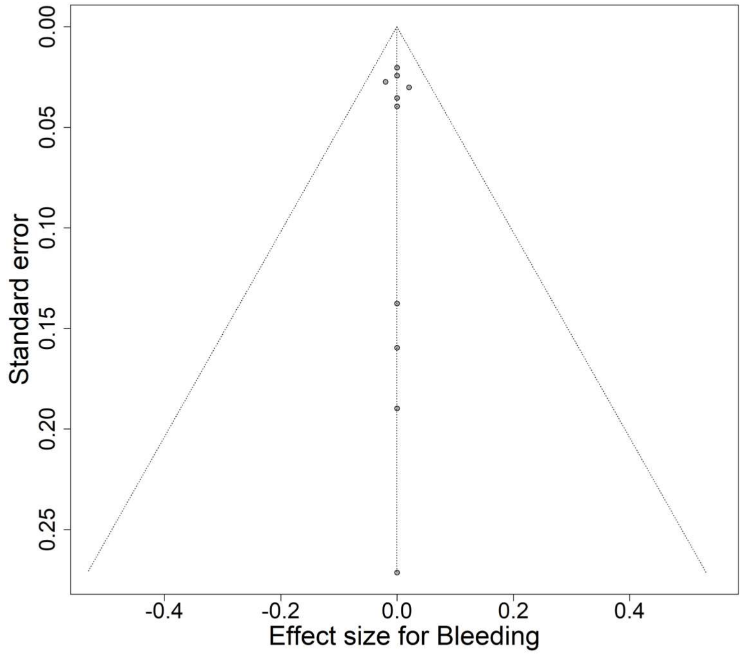

Fig S4. Funnel plot for Bleeding, comparing TBMC with TBNA

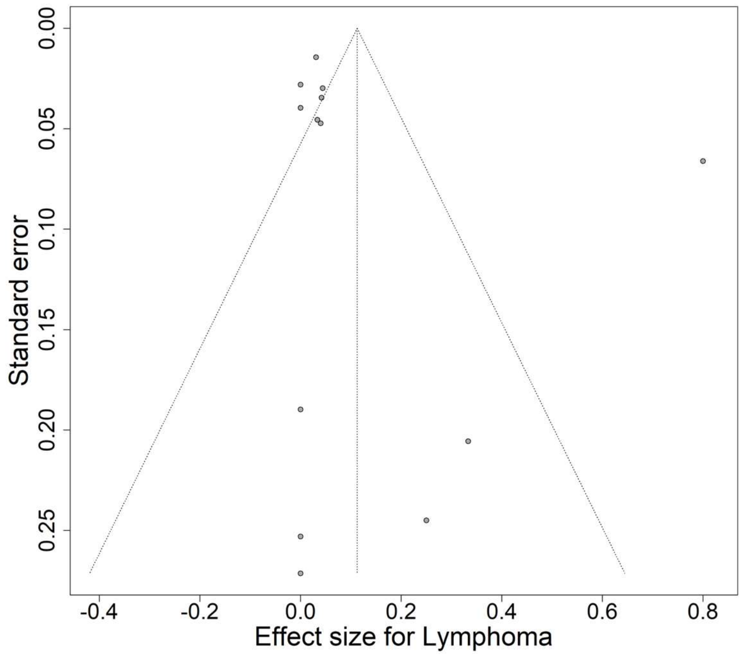

Fig S5. Funnel plot lymphoma

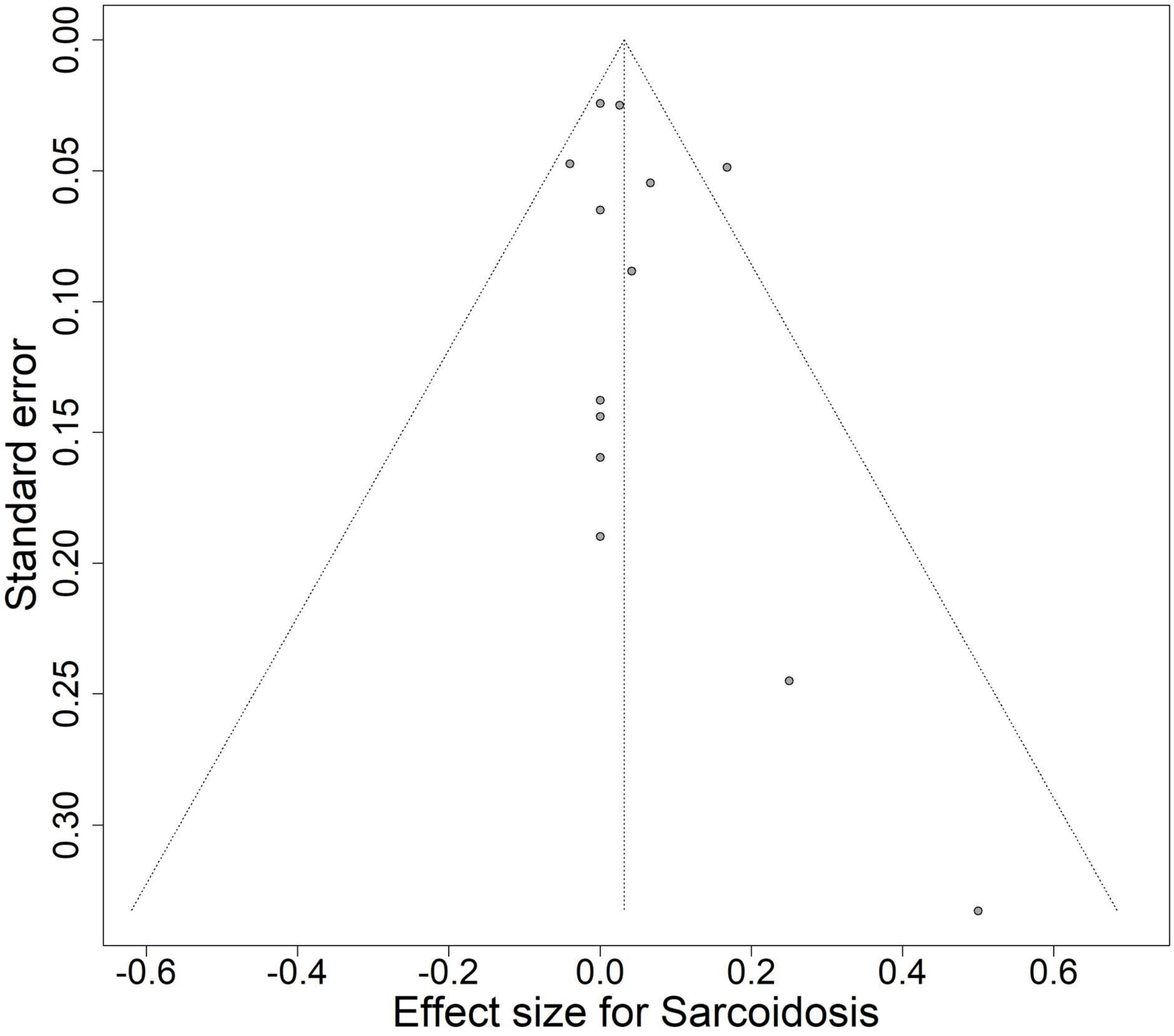

Fig S6. Funnel plot sarcoidosis

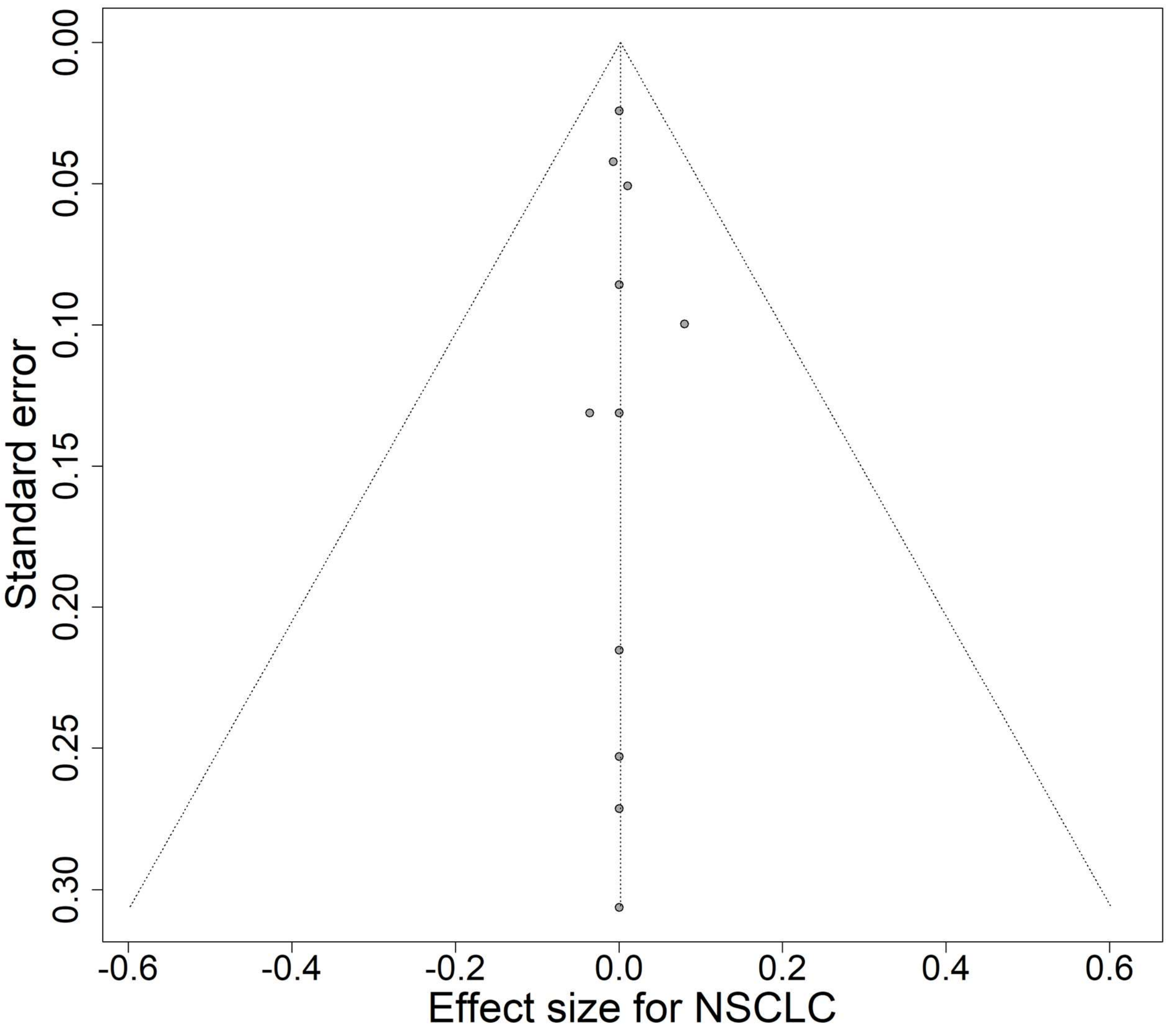

Fig S7. Forrest plot non small cell lung cancer

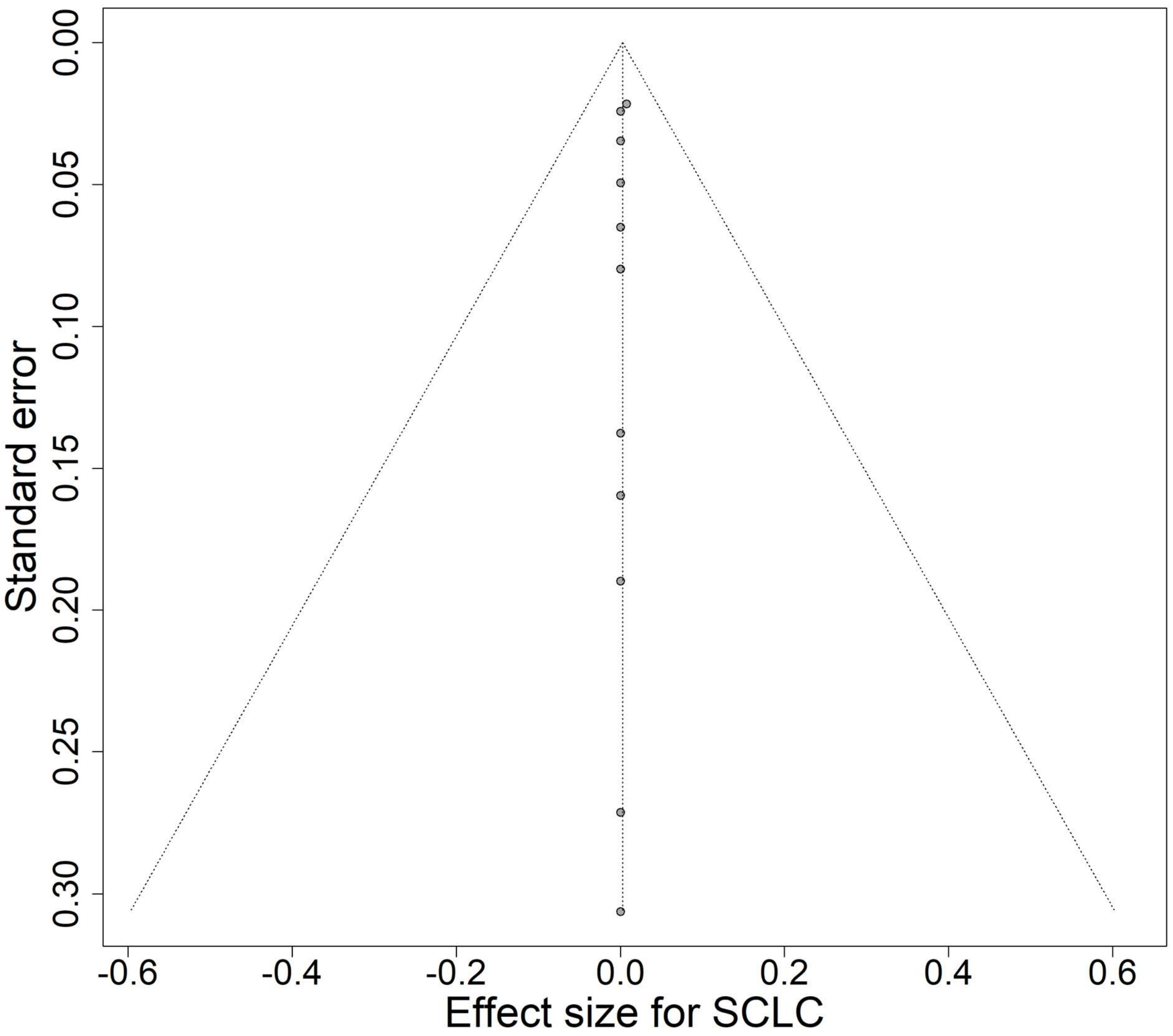

Fig S8. Small cell lung cancer

Criteria for rating the quality of the included studies (Modified QUADAS-2).

Domain 1: Patient selection (mediastinal/hilar lymphadenopathy or masses undergoing EBUS)

Risk of bias

1. **Was a consecutive or random sample of patients enrolled?** *Yes* if consecutive or random sampling was explicitly stated; *No* if convenience/non-consecutive sampling was used; *Unclear* if not reported.
2. **Was a case–control design avoided?**
   - *Yes* if patients were enrolled as a clinical cohort undergoing EBUS for suspected disease; *No* if patients with a known diagnosis were compared with separate controls (case–control design); *Unclear* if not reported.
3. **Did the study avoid inappropriate exclusions?**

- *Yes* if all eligible patients were included; *No* if subgroups likely to affect yield were excluded without justification (e.g., small nodes, specific stations, high-risk patients); *Unclear* if insufficient information was provided.

Concerns regarding applicability

4. **Are there concerns that the included patients and setting do not match the review question?** *No* if adult patients undergoing EBUS for mediastinal/hilar sampling were included; *Yes* if the population/setting was not representative of routine EBUS practice or was highly selective without alignment to the review question; *Unclear* if not reported.

Domain 2: First test (EBUS-TBNA and/or EBUS-TBMC)

Risk of bias

1. **Were the index test results interpreted without knowledge of the other test?**
   - *Yes* if blinding was explicitly stated; *No* if interpretation was clearly informed by the reference standard; *Unclear* if not reported.
2. **Were the criteria for a "diagnostic" result and/or "sample adequacy" pre-specified?**
   - *Yes* if the study predefined diagnostic/adequacy criteria (e.g., diagnostic yield definition, adequacy for histology/IHC/flow cytometry/NGS); *No* if criteria were post hoc, inconsistent between arms, or not clearly defined; *Unclear* if not reported.

Concerns regarding applicability

3. **Are there concerns that the first test, its conduct, or its interpretation differ from the review question?**

- *No* if EBUS-TBNA/TBMC were performed and interpreted using standard techniques and clinically relevant criteria; *Yes* if non-standard procedures or mixed/indistinguishable techniques were used (e.g., combined procedures without separable results); *Unclear* if not reported.

Domain 3: Second test

Risk of bias

1. **Were the second test results interpreted without knowledge of the first test results?**
   - *Yes* if blinding was explicitly stated; *No* if interpretation was influenced by index test results; *Unclear* if not reported.
2. **Were the criteria for a "diagnostic" result and/or "sample adequacy" pre-specified?**
   - *Yes* if the study predefined diagnostic/adequacy criteria (e.g., diagnostic yield definition, adequacy for histology/IHC/flow cytometry/NGS); *No* if criteria were post hoc, inconsistent between arms, or not clearly defined; *Unclear* if not reported.

Concerns regarding applicability

3. **Are there concerns that the target condition as defined by the tests does not match the review question?**

- *No* if final diagnoses (malignant/benign and, when applicable, subtype) were defined using accepted criteria; *Yes* if definitions were inconsistent or not clinically meaningful for the review question; *Unclear* if not reported.

Domain 4: Flow and timing

Risk of bias

1. **Was there an appropriate interval between the first test and the second test?**

- *Yes* if the interval was clinically appropriate (same admission/procedure or within a reasonable timeframe without intervening treatment likely to alter diagnosis); *No* if excessively long or clearly prone to misclassification; *Unclear* if not reported.

2. **Were all enrolled patients included in the analysis?**
   - *Yes* if all patients were accounted for in the analysis ("intention to diagnose"); *No* if patients/specimens were excluded post hoc without justification; *Unclear* if insufficient information was provided.
